# Latent Profiles Based on Light Physical Activity, Sedentary Behavior, Perceived Body Shape, and Body Mass Index in Patients with Dyslipidemia Influence Their Quality of Life

**DOI:** 10.3390/ijerph16204034

**Published:** 2019-10-21

**Authors:** Saengryeol Park, So-Youn Park, Gapjin Oh, In-Hwan Oh

**Affiliations:** 1 Department of Preventive Medicine, School of Medicine, Kyung Hee University, 26, Kyungheedae-ro, Dongdaemun-gu, Seoul 02453, Korea; 2 Department of Medical Education and Humanities, School of Medicine, Kyung Hee University, 26, Kyungheedae-ro, Dongdaemun-gu, Seoul 02453, Korea; 3 Department of Sport Marketing, Kyung Dong University, 27, Kyungdong University-ro, Yangju, Gyeonggido 11458, Korea

**Keywords:** latent profile analysis, perceived body shape, physical activity, sedentary behavior

## Abstract

Despite the increasing prevalence and economic burden of dyslipidemia in South Korea, we have little data on the physical activity of patients. Thus, we aimed to investigate how quality of life among patients with dyslipidemia is influenced by a combination of the following variables: light physical activity (PA), sedentary behavior (SB), perceived body shape, and body mass index (BMI). We examined data from the Sixth Korean National Health and Nutrition Examination Survey (KNHANES VI 2015), collected in 2015 by the Korean Centers for Disease Control and Prevention. The analysis included 534 individuals with dyslipidemia out of 7380 survey participants. Latent profile analysis identified three latent classes of individuals based on their physical profiles. Class 1 patients (active; *n =* 48) were more active, possessed more positive views of their body shape, were less sedentary, and had a lower BMI than Class 3 patients (inactive; *n =* 154). Class 2 patients (moderate; *n =* 331) had profiles in between the other two classes. Additionally, Class 1 and 2 patients had better quality of life than Class 3 patients. Our results suggest that promoting light PA and altering perceived body shape through counselling may improve quality of life in patients with dyslipidemia.

## 1. Introduction

Dyslipidemia is a condition involving abnormal plasma lipid content [1]. It is a major cause of ischemic heart disease [2] and atherosclerosis [3]. Dyslipidemia (total cholesterol: TC ≥5 mmol/L) is highly prevalent in the Americas (47.7%) and Europe (53.7%) [4]. Despite initially occurring at lower rates in South Korea (TC ≥6.22 mmoL = 13.75% in 2010–2012) [5], dyslipidemia prevalence has been gradually increasing in recent years. For example, the number of patients with dyslipidemia increased by 45% from 2014 (*n* = 1,381,385) to 2018 (*n* = 2,007,318) [6]. Medical costs in South Korea have correspondingly increased ($77,488 USD in 2014 to $134,342 USD in 2018) [6]. Because dyslipidemia is associated with body mass [7], some have suggested that lifestyle modifications geared at decreasing body mass, such as increasing physical activity (PA) and reducing sedentary behavior (SB), could potentially alleviate the condition [8].

A few encouraging signs for this association include the finding that light PA (e.g., light walking) decreases dyslipidemia indicators such as TC and high-density lipoprotein (HDL) cholesterol [9,10]. In contrast, SB (e.g., sitting) is linked to elevated triglycerides, HDLs, glucose [11], and other cardiometabolic health markers [12]. Understanding whether patients can be classified based on lifestyle factors would be helpful for researchers to determine how lifestyle influences dyslipidemia incidence.

Quality of life has psychological, physical, and social dimensions [13]. The concept provides us with a measure of a disease’s subjective impact on patients and how they respond to treatment [14]. Therefore, understanding subjective quality of life has become important in health-care settings [15]. In South Korea, dyslipidemia is the fourth most common disease (25.21%) among adults over 50, following hypertension, osteoarthritis, and diabetes mellitus [16]. Additionally, women with dyslipidemia rate their quality of life lower than women without dyslipidemia [17]. However, no previous study has examined what factors correlate with quality of life in patients affected by this condition.

Latent profile analysis (LPA) is a person-centered approach that should be effective for exploring subtypes of patients with dyslipidemia. The method assumes that patients can be classified into latent groups based on a set of characteristics (their profiles) [18]. This perspective is different from the variable-centered nature of regression models, which examine direct associations between predictors and outcomes. Currently, most research on dyslipidemia is variable-centered [17]. However, an emerging body of research is applying LPA to investigate quality of life [19,20], given that the concept comprises of multiple lifestyle and psychological factors [13]. Thus, LPA can be applied to determine how distinct profiles of patients with dyslipidemia are associated with variation in quality of life.

In this study, we aimed to first investigate whether South Korean patients with dyslipidemia can be classified into latent classes based on light PA, SB, BMI, and perceived body shape. Next, we aimed to examine how these latent classes influenced quality of life. Our results should provide insight on key correlates of quality of life among patients with dyslipidemia, thus helping us develop appropriate management methods related to lifestyle changes. Such methods are particularly important among this population because no drug treatments are available to manage mild dyslipidemia [21]. To the best of our knowledge, this is the only study investigating latent relationships between the selected variables and quality of life in patients with dyslipidemia.

## 2. Materials and Methods

### 2.1. Participants and Data Collection

**Figure 1:**
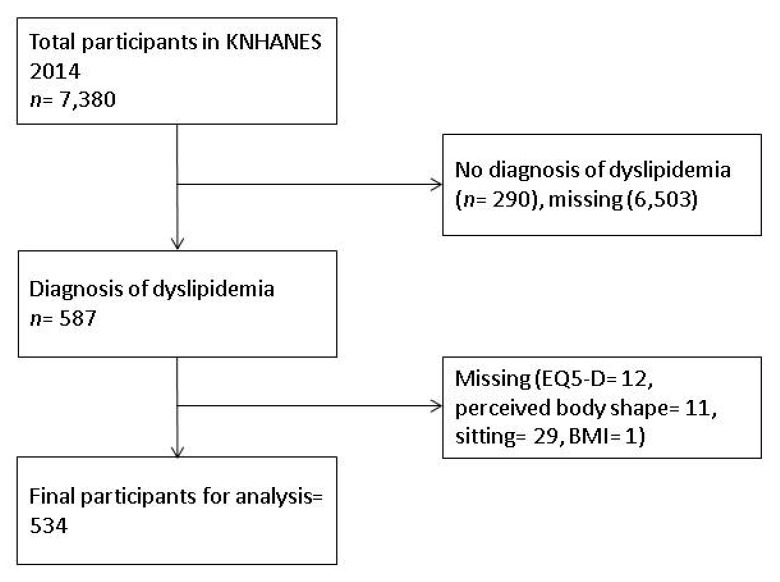
Participants included in the study.

This study analyzed data from the Sixth Korea National Health and Nutrition Examination Survey: KNHANES VI 2015. The survey was performed in 2015 on 7380 nationally representative participants by the Korean Centers for Disease Control and Prevention (KCDCP). After written consent from the participants was obtained, trained medical and research staff conducted health examinations (BMI), self-reports (e.g., educational background, social economic status), and interviews (PA, SB) to measure health-related variables [22]. Data collection occurred at a mobile center of each primary sampling unit. Detailed information on the procedures was published previously [22]. This study selected participants who took drugs for dyslipidemia (*n* = 534; see Figure 1). The research was approved by the ethical committee board of a Korean university (No. 2018-03-012-001).

### 2.2. Variables

#### 2.2.1. Light PA

We used two items from the International Physical Activity Questionnaire (IPAQ) [23] to assess this variable. The questionnaire instructed participants to record the frequency ‘*How many days during the past 7 days did you walk for at least 10 min at a time?*’ and time they spent in light PA ‘*How much time did you usually spend walking on one of those days?*’. Data were represented as means (min/d).

#### 2.2.2. Sedentary Behavior

We assessed this variable using items from the Korean version of the Global Physical Activity Questionnaire (GPAQ) [24]. Participants were instructed to record the total time they spent sitting/reclining each day in min/d ‘*How much time do you usually spend sitting or reclining on a typical day?*’. Examples of SB were sitting or watching TV.

#### 2.2.3. Perceived Body Shape

We asked participants to answer the question, “*How do you feel about your body shape?*” [25], using a Likert scale (1 = ‘very lean’ to 5 = ‘very obese’). High scores indicate that patients perceived themselves as highly obese.

#### 2.2.4. Body Mass Index

To calculate BMI, we divided body weight (Seca 225) by squared height (kg/m^2^; GL-6000-20; Cass Korea, Seoul, South Korea).

#### 2.2.5. Quality of Life

This variable was assessed using five items from the Korean version of the EuroQoL five-dimensional questionnaire (EQ-5D) [26] on a 3-point Likert scale. The questions involved mobility (M; 1 = ‘*I have no problems walking about*’, 3 = ‘*I am unable to walk about*’; self-care (SC; 1 *=* ‘*I have no problems washing or dressing myself*’, 3 = ‘*I am unable to wash or dress myself*’); ability to engage in usual activities (UA; 1 = ‘*I have no problems doing my usual activities*’, 3 = ‘*I am unable to do my usual activities*’); physical discomfort (PD; 1 = ‘*I have no pain or discomfort*’, 3 = ‘*I have extreme pain or discomfort*’); and anxiety/depression (AD; 1 *=* ‘*I am not anxious or depressed*’, 3 *=* ‘*I am extremely anxious or depressed*’). Responses were weighted for conversion to an EQ-5D index based on a previously published formula [26]:

$$\begin{array}{ll} \mathrm{EQ}5D\;\mathrm{index}\;= & 1-(0.05\;+\;0.096\;\times \;M2\;+\;0.418\;\times \;M3\;+\;0.046\;\times \;\mathrm{SC}2\\ & +\;0.136\times \;\mathrm{SC}3+\;0.051\;\times \;\mathrm{UA}2\;+\;0.208\;\times \;*\mathrm{UA}3\\ & +\;0.037\;\times \;\mathrm{PD}2+\;0.151\;\times \;\mathrm{PD}3\;+\;0.043\;\times \;*\mathrm{AD}2\\ & +\;0.158\;\times \;\mathrm{AD}3\;+\;0.05\;\times \;N3) \end{array}$$

Weighting was performed using the following criteria: M2 “level 2” = 1, otherwise = 0; M3 “level 3” = 1, otherwise = 0; SC2 “level 2” = 1, otherwise = 0; SC3 “level 3” = 1, otherwise = 0; UA2 “level 2” = 1, otherwise = 0; UA3 “level 3” = 1, otherwise = 0; PD2 “level 2” = 1, otherwise = 0; PD3 “level 3” = 1, otherwise = 0; AD2 “level 2” = 1, otherwise = 0; AD3 “level 3” = 1, otherwise = 0; N3 “level 3” = 1, otherwise = 0. This conversion resulted in higher scores representing better quality of life.

#### 2.2.6. Covariate 

Alcohol consumption of individuals over the past year was categorized into two groups (drink = 1, no drink = 0) [27]. Additionally, smoking status was separated into current smoker = 2, past smoker = 1, non-smoker = 0 [28].

### 2.3. Statistical Analysis

Participants were classified according to activity level through LPA [29]. First, all profiles were standardized. Next, a maximum likelihood estimator was adopted because it is robust for non-normal distribution. To obtain properly converging models, optimizations were increased from starting values of 500 and 20 to 600 and 120, then to 600 and 160. The final model was selected using Akaike Information Criterion (AIC) [30], Bayesian Information Criterion (BIC) [31], sample-size adjusted BIC (SSA-BIC) [32], and bootstrapped likelihood ratio test (BLRT) [33]. High AIC, BIC, and SSA-BIC values indicated a better fit. A significant BLRT value indicated that the current number (*k*) of classes was better than *k*-1 classes. Drinking and smoking were used as covariates across all analyses to determine latent profiles, thus controlling for other lifestyle factors. Thus, participants in the same class had homogeneous characteristics of light PA, SB, BMI, and perceived body shape. A further analysis then compared associations between latent classes and an outcome variable (auxiliary option) [34]. Effect size (Cohen’s *d*) was estimated for each group. Statistical significance was set at *p* < 0.05. Analyses were performed in SPSS version 22 (IBM Corp, Armonk, NY, USA, 2012) and Mplus version 7.3 (Muthén and Muthén, Los Angeles, CA, USA).

## 3. Results

Participants were between 27–80 years old (mean = 63.65 ± 10.02, 34.83% female). Average BMI was 25.02 ± 3.35 kg/m^2^ (see Table 1). Average systolic and diastolic blood pressure was 124.65 mm Hg and 74.43 mm Hg, respectively. A large number of participants had only completed elementary school (*n* = 228, 42.70%). Overall, participants spent 452.7 min/d in SB and 38.71 min/d engaging in light PA.

We identified negative correlations between SB and light PA, light PA and BMI, perceived body shape and quality of life, as well as BMI and quality of life (see Table 2). Additionally, light PA and quality of life, along with body shape and BMI, were positively correlated.

Models with three classes had lower Akaike Information Criterion (AIC), Bayesian Information Criterion (BIC), and sample-size adjusted BIC (SSA-BIC) values than models with one class (Table 3). Models with two and three classes exhibited significant bootstrapped likelihood ratio test (BLRT) values. In the two-class model, participant percentages in each class were 90% and 11%; in the three-class model, percentages were 9%, 62%, and 29%. After accounting for these best-fit indices, we selected the three-class model for further analyses. Latent profile analysis did not detect proper four-class models despite step-by-step increases of starting values.

**Figure 2:**
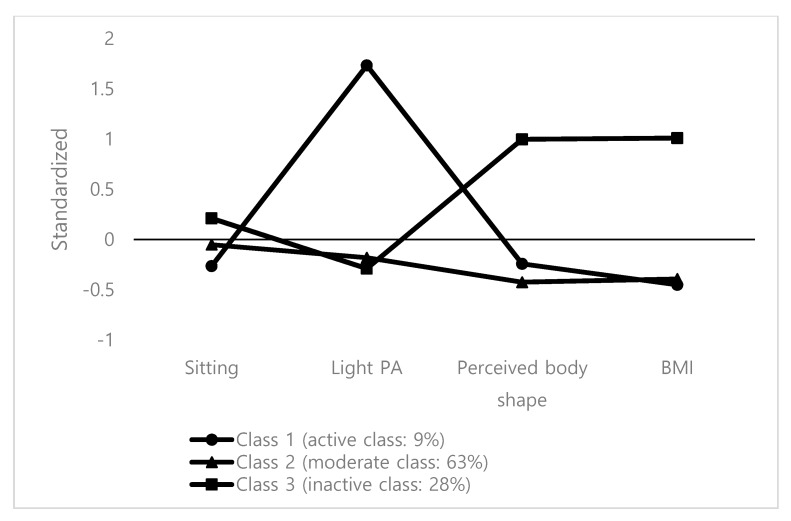
Latent profiles of sedentary behavior, light physical activity (PA), perceived body shape, and BMI.

We assigned names to each class based on profile characteristics (active class: *n* = 48, 9%; moderate class: *n* = 331, 62%; inactive class, *n* = 154, 29%; Table 4 and Figure 2). Time spent in SB and BMI increased from active to inactive classes, while time spent in light PA decreased. Inactive individuals had the worst perception of their own body shape. Based on Cohen’s *d* thresholds (small effect size = 0.2–0.3, medium = 0.5, large ≥0.8) [35], light PA had large effect sizes (Cohen’s *d*_active-moderate_ = 0.80, Cohen’s *d*_active-inactive_ = 0.81), perceived body shape (Cohen’s *d*_active-inactive_ = 0.67, Cohen’s *d*_moderate-inactive_ = 0.52) and BMI (Cohen’s *d*_active-inactive_ = 0.57, Cohen’s *d*_moderate-inactive_ = 0.50) had medium effects, while SB had small effects (Cohen’s *d* = 0.14–0.18).

Quality of life decreased from the active to the inactive class (Table 5). Both active (*d*_active-inactive_ = 0.49) and moderate classes (*d*_moderate-inactive_ = 0.26) had significantly higher values than the inactive class.

## 4. Discussion

Here, we successfully used LPA to identify three latent classes based on SB, light PA, perceived body shape, and BMI in South Korean patients with dyslipidemia. Our results have important underlying implications for individual health. Specifically, light PA plays an important role in classifying patients with dyslipidemia. Additionally, perceived body shape is influential in increasing quality of life. Ours is the first attempt to examine whether potential contributors to quality of life among South Korean patients with dyslipidemia could be classified into latent classes. The profiles we identified provide evidence for the roles of light PA and perceived body shape in managing quality of life, knowledge that should be beneficial to healthcare practitioners.

Our results suggest that health professionals should focus on promoting light PA as intervention. We found large effect sizes in light PA when discriminating between active patients and those grouped in the less active classes. Active patients engaged in roughly seven and six times more light PA than inactive and moderate patients, respectively. Indeed, light PA is the most frequently recommended form of activity [36] because data indicate that it is both easy to perform and effective for managing dyslipidemia-related variables [9]. Furthermore, an isotemporal substitution approach (i.e., replacing SB with moderate-to-vigorous PA) increased quality of life in older adults [37]. One way to encourage PA increases is altering the environment. For example, several characteristics in urban areas have successfully motivated people to engage in PA regularly: safety features (lights, even surfaces), aesthetics (green space, waterfalls), and separate pedestrian areas [38]. Thus, we encourage future studies to empirically develop intervention and alter environments to increase light PA as a method to enhance quality of life.

Besides light PA, perceived body shape should be viewed as a key factor influencing quality of life. Effect sizes of perceived body shape were comparable to BMI effect sizes, and the latter is a well-established correlate of dyslipidemia [39,40]. Indeed, a previous study found that perceived body shape might be a more important risk factor for depression than BMI [41]. Perceived body shape could be improved successfully through intervention, including a supervised PA program [42]. Thus, a viable strategy worth investigation in the future is combining light PA (e.g., brisk walking) with psychological counselling to improve quality of life in South Koreans.

A limitation of this cross-sectional study is that we were unable to address any causality. Thus, we encourage further cohort and intervention studies to investigate how PA and perceived body shape alters quality of life in these contexts. Another limitation is that our study only examined patients with dyslipidemia and lacks generality. We therefore recommend caution in interpreting the results. Although numerous factors contribute to quality of life, PA and SB are widely investigated variables in research on individuals with a clinical diagnosis [43]. In this study, we addressed how perceived body shape could influence quality of life along with BMI. Thus, our work broadens existing research on quality of life among patients with metabolic disorders, but we acknowledge that the inclusion of even more variables could affect our findings. Further studies including physical function and other lifestyle factors (e.g., more varied levels of PA) would fill gaps existing in our study. In particular, further studies should include moderate-to-vigorous PA, which may be beneficial for quality of life. The use of BMI is another limitation of this study because the measure has well-known limitations as a proxy for obesity [44]. However, a previous study showed perceived body shape is negatively associated with BMI [45], supporting our rationale for examining perceived body shape and BMI simultaneously in LPA. Nevertheless, we encourage future studies to use a measure that better reflects obesity, such as waist circumference. A final limitation is the use of a one-item scale to measure light PA, SB, and perceived body shape. However, the items we selected have been widely used in various population groups and are considered valid in the field [23,24,25,26]. Our decision to use them here allows for easier comparisons between studies.

## 5. Conclusions

For the first time, this study demonstrated that light PA, perceived body shape, and BMI are linked factors influencing quality of life. In particular, our findings indicate that more attention should be paid to perceived body shape as a contributor to quality of life in South Koreans. A priority for healthcare specialists interested in patient quality of life is to ensure the development of appropriate light-PA programs and counselling strategies to elevate body-shape perception.

## Figures and Tables

**Table 1:** Descriptive data of survey participants (*n* = 534).

Variables	Mean (SD)	Range
Age (years)	63.65 (10.02)	27–80
BMI (kg/m^2^)	25.02 (3.35)	15–40
Blood pressure (mm Hg)		
Systolic blood pressure	124.65 (15.41)	87–183
Diastolic blood pressure	74.43 (9.78)	49–105
	*n* (%)	
Gender		
Male	348 (65.17)	
Female	186 (34.83)	
Annual household income		
Low (1)	134 (26.03)	
Middle/low (2)	153 (28.65)	
Middle/high (3)	114 (21.35)	
High (4)	124 (23.22)	
Missing	4 (0.75)	
Education		
Elementary school	228 (42.70)	
Middle school	73 (13.67)	
High school	136 (25.47)	
≥University	96 (17.98)	
Missing	1 (0.19)	
Alcohol consumption status		
Drink	319 (59.74)	
No drink	215 (40.26)	
Smoking status		
Current smoker	355 (66.48)	
Past smoker	119 (22.28)	
Non-smoker	60 (11.24)	

Note: BMI = body mass index.

**Table 2:** Bivariate correlation analyses of study variables.

Variables	2. Light Physical Activity	3. Perceived Body Shape	4. BMI	5. Quality of Life	6. Alcohol Consumption Status	7. Smoking Status
1. Sedentary behavior (sitting)	−0.109 *	0.045	0.048	−0.072	−0.024	0.087 *
2. Light physical activity		−0.035	−0.144 **	0.120 **	0.047	0.005
3. Perceived body shape			0.692 **	−0.078	0.081	−0.020
4. BMI				−0.156 **	0.038	0.034
5. Quality of life					0.178 **	0.052
6. Alcohol consumption status						0.272 **
7. Smoking status						

Note: * = *p* < 0.05, ** = *p* < 0.01; BMI = body mass index.

**Table 3:** Fit statistics of latent profile analysis.

Fit Statistics	1 Class	2 Classes	3 Classes	4 Classes
AIC	9099.53	5348.23	5138.27	N/A
BIC	9150.87	5429.42	5266.63	
SSA-BIC	9112.78	5369.11	5171.40	
BLRT *p*-value		<0.001	<0.001	
Percent of participants per Class (%)		*n* = 477 (90%), *n* = 56 (11%)	Class 1 (*n* = 48: 9%), Class 2 (331: 62%), Class 3 (154: 29%)	

Note: AIC = Akaike Information Criterion, BIC = Bayesian Information Criterion, SSA-BIC = sample-size adjusted BIC, BLRT = bootstrapped likelihood ratio test.

**Table 4:** Unstandardized profile characteristics of the three-class model describing patients with dyslipidemia.

Variables	Active Class (Class 1; *n* = 48, 9%)		Moderate Class (Class 2; *n* = 331, 62%)		Inactive Class (Class 3; *n* = 154, 29%) Inactive		Cohen’s *d* Effect Size		
	M	SD	M	SD	M	SD	*d*_active-moderate_	*d*_active-inactive_	*d*_moderate-inactive_
Sitting	395.60	225.08	441.63	283.49	495.85	478.37	0.18	0.27	0.14
Light PA	153.20	209.13	26.69	80.91	19.74	100.42	0.80	0.81	0.08
Perceived body shape	3.24	1.98	3.06	3.35	4.41	1.58	0.07	0.67	0.52
BMI	23.50	6.21	23.69	8.02	28.38	10.52	0.03	0.57	0.50

Note: Light PA = light physical activity.

**Table 5:** Between-class comparisons in quality of life.

Classes	Quality of Life	
	Mean	SD
Active class (class 1; *n* = 48)	0.95	0.14
Moderate class (class 2; *n* = 331)	0.91	0.16
Inactive class (class 3; *n* = 154)	0.86	0.22
Class comparison	Chi square	*p*
Overall test	11.64	0.003
Active vs. moderate	2.99	0.084
Active vs. inactive	11.41	0.001
Moderate vs. inactive	5.44	0.019
Cohen’s *d* effect size		
*d*_active-moderate_	0.27	
*d*_active-inactive_	0.49	
*d*_moderate-inactive_	0.26	
